# Flower color preferences of insects and livestock: effects on *Gentiana lutea* reproductive success

**DOI:** 10.7717/peerj.1685

**Published:** 2016-03-15

**Authors:** Mar Sobral, María Losada, Tania Veiga, Javier Guitián, José Guitián, Pablo Guitián

**Affiliations:** 1 Departamento de Bioloxía Celular e Ecoloxía/Facultade de Bioloxía, Universidade de Santiago de Compostela, Santiago de Compostela, Spain; 2 Departamento de Botánica/Facultade de Bioloxía, Universidade de Santiago de Compostela, Santiago de Compostela, Spain

**Keywords:** Flower color variation, *Gentiana lutea*, Insect herbivory, Large mammal herbivory, Herbivore preferences

## Abstract

Angiosperms diversification was primarily driven by pollinator agents, but non-pollinator agents also promoted floral evolution. *Gentiana lutea* shows pollinator driven flower color variation in NW Spain. We test whether insect herbivores and livestock, which frequently feed in *G.lutea*, play a role in *G. lutea* flower color variation, by answering the following questions: (i) Do insect herbivores and grazing livestock show flower color preferences when feeding on *G. lutea*? (ii) Do mutualists (pollinators) and antagonists (seed predators, insect herbivores and livestock) jointly affect *G. lutea* reproductive success? Insect herbivores fed more often on yellow flowering individuals but they did not affect seed production, whereas livestock affected seed production but did not show clear color preferences. Our data indicate that flower color variation of *G. lutea* is not affected by insect herbivores or grazing livestock.

## Introduction

Despite pollinators being considered the principal driver of floral diversification and speciation (Bradshaw & Schemske, 2003; Herrera, Castellanos & Medrano, 2006; Whittall & Hodges, 2007; Van der Niet, Peakall & Johnson, 2014), non-pollinator agents also interact with plants and promote floral evolution (Strauss & Whittall, 2006; Andersson, 2008). This is the case with antagonist animals, such as herbivores, which damage plants as a result of resources extraction (Strauss & Whittall, 2006; Whittall & Carlson, 2009), affecting fitness and, therefore, potentially causing natural selection on plant traits (Wise & Hébert, 2010; Agrawal et al., 2012; De Jager & Ellis, 2014).

Plant reproductive success can be shaped by the balance between mutualistic and antagonistic interactions which may maintain floral trait variation (Herrera et al., 2002; Lavergne, Debussche & Thompson, 2005; McCall & Irwin, 2006; Bartkowska & Johnston, 2012). Herbivory can have a negative effect on plant fitness in a greater or lesser extent depending on pollinator presence or absence (Herrera, 2000; Herrera et al., 2002), and the positive effect exerted by pollinators may depend on herbivore presence (Gómez, 2003; Gómez, 2005a). Thus, phenotypic variation of a floral trait, such as flower color, can result from the balance between the positive selection pressures exerted by pollinators and seed dispersers, and the negative selection pressures exerted by herbivores or seed predators (Herrera, 2000; Herrera et al., 2002; Irwin et al., 2003; Asikainen & Mutikainen, 2005; Frey, 2004; Strauss, Irwin & Lambrix, 2004; Andersson, 2008; Bartkowska & Johnston, 2012). Selective pressures exerted by abiotic factors (Galen, 2000; Warren & Mackenzie, 2001; Streisfeld & Kohn, 2007) and historical processes or genetic drift (Mitchell-Olds, Willis & Goldstein, 2007) may also influence phenotypic variation.

Invertebrate herbivory (mainly insects and gastropods) negatively affects plant fitness causing natural selection on plant traits, including floral traits, such as flower color (Fineblum & Rausher, 1997; Frey, 2004; Whittall & Carlson, 2009; Bartkowska & Johnston, 2012). Additionally, vertebrate herbivory (typically represented by large mammal herbivores) affect plant fitness and community composition (Herrera, 1984; Agustine & McNaughton, 1998; Knight, 2003; Gómez, 2005b; Allsup, 2014). Interaction with mammals can also have a positive effect for the plants. Reproductive success of many flowering plant species relies on browsing ungulates activity, since they play an essential role if ungulates act as seed dispersal agents within plant communities (for example, Herrera, 1984; Herrera, 2000). Thus, the effect of mammal herbivory can be either positive, if they act as seed dispersal agents, or negative if they act as herbivores. Many plant communities interact not only with wild fauna but also with domesticated mammals. Domesticated ungulates such as cattle, horses and sheep often feed on wild plant populations (for example during transhumance practices) potentially exerting natural selection on them.

The type and strength of selection exerted on plant attributes depend on herbivore preferences during foraging (Agustine & McNaughton, 1998; Asikainen & Mutikainen, 2005; Agrawal, Lau & Hambäck, 2006). Livestock could discriminate between flower color morphs (Phillips & Lomas, 2001), and thus might be able to show color preferences. But, even if livestock could not detect flower color differences, it still may differentially feed on a particular color morph if it shows preferences for any trait correlated with flower color (see Lande & Arnold, 1983). As it could be the case for some herbivory defenses which are known to be correlated with floral pigments (Simms & Bucher, 1996).

The effect of livestock (negative or positive) is expected to be stronger than the exerted by insect herbivores or pollinators due to the amount of damage or seed dispersal that large vertebrates are able to exert. Thus, it is necessary to take their effect into account in order to have a holistic view of the biotic forces exerting selective pressures on traits such as floral color (Johnson, Campbell & Barrett, 2015).

Although it has been long recognized that both pollinators and herbivores play an important role on plant evolutionhttp://brahmaputra:8080/tf/texfolio/APP/connector/0/791/source/peerj1685-main14580233070791.png (Ehrlich & Raven, 1964), their effects are usually studied independently and under different scopes (Johnson, Campbell & Barrett, 2015). Moreover, the effects of grazing mammals on flower traits have been disregarded (but, see Juenger & Bergelson, 1997; Gómez, 2003; Gómez et al., 2009; Ågren et al., 2013), and the potential effects of domesticated mammal herbivores on floral traits are unknown. Here we explore for the first time the simultaneous effect of wild and domesticated animals on plant traits, particularly regarding their potential effect on flower color variation.

*Gentiana lutea* flower color varies continuously from orange to yellow within and among populations in NW Spain (Sobral et al., 2015)—where livestock interacts with plant communities (see Blanco-Fontao, Quevedo & Obeso, 2011) and commonly feed on *G. lutea*. This corolla color variation has a genetic basis (Zhu et al., 2002; Zhu et al., 2003) and is not related to abiotic factors such as radiation, altitude, temperature or rainfall (Veiga et al., 2015a). Two flower color varieties are described (Laínz, 1982; Renobales, 2003) and a partial hybridization barrier exists between the yellow *G. lutea* var. *aurantiaca* and the orange *G. lutea* var. *lutea* (Losada et al., 2015). *G. lutea* is strongly dependent on pollinators which, together with seed predators, show flower color preferences causing selection on flower color (Losada et al., 2015; Veiga et al., 2015b).

Here we test if insect herbivores—mainly adults belonging to Orthopthera and Coleoptera orders, and larvae from different insect groups—and large mammal domesticated herbivores (local extensive livestock, mainly cows and horses) affect flower color variation in *Gentiana lutea*—while having into account the effect of pollinators and seed predators. For this purpose, the following questions were formulated: (i) Do insect herbivores and livestock show flower color preferences when feeding on *G. lutea*? (ii) Do mutualists (pollinators) and antagonists (seed predators, insect and livestock) jointly affect *G. lutea* reproductive success?

## Materials & Methods

### Study area

**Figure 1:**
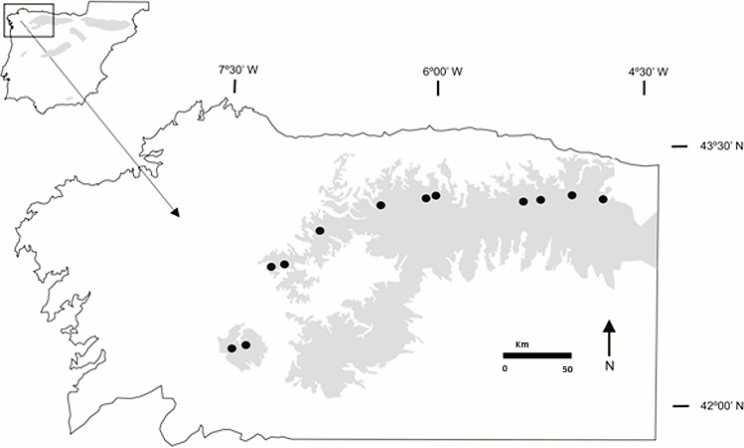
Location of the *Gentiana lutea* populations sampled. The shaded region indicates the distribution of *G. lutea* in the Cantabrian Mountains, NW Spain. Black dots represent the 12 studied populations (from W to E): San Mamede, Queixa, Loureses, O Cebreiro, Os Ancares, Leitariegos, Torrestío, Ventana, San Isidro, Señales, Pontón and San Glorio.

Our study area covered the distribution of *Gentiana lutea* in the Cantabrian Mountains, NW Spain (see Fig. 1). In 2010, we visited 8 populations (Cebreiro, Ancares, Leitariegos, Torrestío, Ventana, San Isidro, Señales and San Glorio) and 12 in 2011 (Queixa, San Mamede, Loureses and Pontón were new). All studied populations were haphazardly selected along a 230 km longitudinal gradient from the San Mamede population (42°12′N, 7°30′W; at the western limit) to the San Glorio population (43°04′N, 4°45′W; at the eastern limit), and localized at high altitudes from 1,100 m to 1,700 m.a.s.l., on grassy pastures and hillsides used extensively by local livestock. For this research, we received a field permit from the Environmental Territorial Service of León, Territorial Delegation of Government of Spain, Regional Government of Castilla and León (ID:12_LE_325_RNA_PuebladeLillo_INV; Reference: 06.01.013.016/ROT/abp; File number: AEN/LE/103/12).

### Plant species

*Gentiana lutea* (*Gentianaceae*) is a rhizomatous perennial herb distributed throughout central and southern European mountains, living at montane and sub-alpine levels (approximately from 700 to 2,000 m.a.s.l.) and mainly associated with livestock grazing grasslands (Hesse, Rees & Müller-Schärer, 2007; Anchisi et al., 2010). This is a long-lived geophyte, which usually develops one unbranched stout stem (rarely two or three) measuring up to 190 cm tall; and shows a basal rosette formed from lanceolate-elliptic leaves measuring 190–350 × 55–150 mm (Renobales, 2012). Fertile stems bloom in summer (June–July), and show several tens of bisexual and actinomorphic flowers grouped in pseudo-whorls. Flowers present a bicarpellate ovary fixed over a split calix, a stigma with two lamellae and (4–8) petals fused on the basis. Corollas have an open structure, which facilitates pollinator access (mainly insects belonging to Hymenoptera and Diptera orders) to flower nectaries. Corolla color varies from orange to yellow along the *G. lutea* distribution range in the Cantabrian Mountains, Spain (Sobral et al., 2015). *G. lutea* fruits are capsules, which hold a great number of elliptic, flattened and winged seeds, measuring 2.5–4.5 mm, which ripen in summer (Renobales, 2012). Wind is the main seed dispersal agent (Struwe & Albert, 2002). *G. lutea* may be considered a toxic species, because it contains relatively high levels of herbivory deterrents (Smit, Ouden & Müller-Schärer, 2006; Hesse, Rees & Müller-Schärer, 2007).

### Field procedures

#### Plant traits measurement

In July 2010, during blooming, we measured flower color on ten randomly chosen flowers per plant. In 2011, we measured color on only three flowers per plant because we found that the coefficient of variation for flower color within plants was asymptotic, reaching a plateau after three flowers. Each petal was measured three times. Final color spectrum data for individual plants came from the mean of these three measurements per petal for 10 petals belonging to 10 different flowers in 2010 (30 floral color data per plant in 2010) and three measurements for each of three petals belonging to three different flower in 2011 (nine color data per plant in 2011). We measured floral color in a total of 2,711 flowers belonging to 504 plants across 12 *G. lutea* populations. With the aim of taking into account indirect selection on correlated plant traits (see Veiga et al., 2015b), we also measured the stalk height (the height of the stalk from the base of the plant to the top, in cm) and the leaf length (the length of the longest basal leaf from the insertion to the tip, in mm) in each plant.

Flower color was measured by means of a spectrometer (USB2000+; Ocean Optics, Inc., Dunedin, FL, USA) and the petal color spectra were processed using the SpectraSuite^®^ software (Ocean Optics, Inc., Dunedin, FL, USA). No differences among populations and between color morphs (orange or yellow colors, discernible by human eye) were found in the UV light range in a previous study (see Veiga et al., 2015b). Thus, flower color variation was described by means of the CIELab Colorimetric System (International Commission on Illumination, 2004). This colorimetric system is based on the visible light range of the electromagnetic spectrum and allows for a transformation of the measured reflectance spectrum into three variables, which describe the flower color variation: *L* (brightness of color, from black to white), *a* (red color variation, from green to red) and *b* (yellow color variation, from blue to yellow; see Veiga et al., 2015b for more information on the flower color measurements).

These three flower color variables (*L*, *a*, and *b*) were reduced by principal component analysis (PCA). The first principal component (PC1) explained 63% of variance of a, *b* and *L*; thus, PC1 was used as the flower color variable in the statistical analyses. Correlations between the original color variables and PC1 show that low scores in PC1 indicate orange colors and high scores indicate yellow colors.

#### Insect herbivory

A total of 162 individual plants were randomly selected before blossom started (June) in each of 8 populations studied in 2010 (Cebreiro, Ancares, Leitariegos, Torrestío, Ventana, San Isidro, Señales and San Glorio). The insect herbivory percentage was quantified visually for all leaves of each plant during flowering (July). For this, we used the scale designed by Dirzo & Domínguez (1995), which establishes six categories: the first category includes leaves without damage; the second, leaves with 1–6% of damage; the third, leaves with 6–12% of damage; the fourth, 12–25% of damage; the fifth, 25–50% and the sixth, 50–100% of damage. With frequencies of leaves in each category of damage, we calculated the index of herbivory per plant: *IH* = (Σ*ni*^∗^*i*)∕*N*; where *n* is the number of leaves in a category, *i* is the category number and *N* is the number of leaves per plant.

#### Livestock herbivory

Livestock consume parts of the plants including leaves, flowers or fruits and parts of the stalk. First, we tested whether the effect of livestock was a negative herbivory effect or, on the other hand, whether livestock could be considered a *G. lutea* seed disperser. We examined livestock herbivory during the fruits ripening season (August) establishing two categories: without evidence of livestock herbivory (0 or absence) and with evidence of livestock herbivory (one or presence) in 183 plants chosen in 8 populations in 2010 and 288 plants chosen across 12 populations in 2011.

Livestock was observed feeding on *G.lutea* at the time that it was bearing fruits. During that time (August 2010 and 2011), we collected 63 livestock fecal samples in all 12 *G. lutea* populations and examined them (40 belonging to cows, 20 to horses and, 3 to sheep). We manually inspected the fecal samples in the lab (3 grams per sample) searching for seed presence within livestock pellets. *G. lutea* seeds found within livestock fecal samples were later examined for germinability. Seeds were distributed on filter paper in petri plates. The state of germination and wetting of the plates were examined on alternate days; the filter paper was removed every 2–3 days to reduce fungal infection. Germination trials lasted for 60 days. Note that we had previously found that after 45 days, 25% of viable *G. lutea*’s seeds were germinated (Losada et al., 2015).

#### Effect of mutualist and antagonist interactions on seed production

When fruits were not yet opened, total number of fruits was counted per plant. Afterwards, 20 fruits were haphazardly collected per individual. Number of viable seeds was counted in each fruit sampled (over 150,000 seeds were counted). *G. lutea* plants set on average 79 fruits, each with a mean of 63 seeds (Sobral et al., 2015). Thus, each plant sets approximately 5,000 seeds on average; hence total number of seeds set per plant was impractical to count. Total seed number per plant was estimated by multiplying the number of fruits per plant times the average number of viable seeds per fruit. In this study, total seed number (reproductive output) was used as a proxy for plant fitness.

With the aim of understanding the effect that insect and livestock herbivory have on the *G. lutea* reproductive output, previously published data on pollination success and seed predation of the same marked plants and reproductive seasons (Sobral et al., 2015) were used to incorporate the effects of these ecological interactions into the models. Note that these models used data from eight populations studied in 2010 (Cebreiro, Ancares, Leitariegos, Torrestío, Ventana, San Isidro, Señales and San Glorio) because we did not collect data on insect herbivory in 2011. See Sobral et al. (2015) for methods to recording pollination and seed predation.

### Statistical analyses

Analyses were performed using the SPSS for Windows, version 20.0 (IBM Corp, 2011). Error distribution, link function and model’s structure were chosen by means of the AICc criterion starting with saturated models (Burnham & Anderson, 2002).

#### Insect preferences for flower color

In order to study the insect preferences for flower color, we analyzed the data from 104 plants from the 8 populations studied in 2010. We used a generalized linear model (GzLM) in which the explanatory variables were population, flower color, stalk height, leaf length, the flower color * stalk height interaction and the flower color * leaf length interaction; and the per-plant percentage of herbivory was the response variable (Table 1). Note that the herbivory index was transformed into a per-plant percentage of herbivory before analysis and was fitted to a Poisson distribution with a log link function. Population was included as a fixed factor into the models since they were selected following a longitudinal gradient. Additionally, the same model (without the population effect) for each of the studied populations was performed (see Appendix S1).

**Table 1:** GzLM fitted to analyze insect herbivory (percentage of herbivory per plant) and its relationship with flower color and other correlated plant traits, such as stalk height and leaf length. *N* = 104 individuals. The statistically significant effects are marked in bold (*P* < 0.05).

Dependent variable	Factor	Wald Chi-Square	d.f.	*P*
Insect herbivory	**Flower color**	3.876	1	**0.049**
	LL	1.646	1	0.200
	SH	1.013	1	0.314
	LL * Flower color	0.498	1	0.481
	SH * Flower color	4.887	1	**0.027**
	Population	12.592	7	0.083

**Notes.**

Factor codes LL Leaf length (mm) SH Stalk height (cm)

#### Livestock preferences for flower color

Some populations present livestock but others do not and populations differ in the average corolla color (Sobral et al., 2015). Therefore, the flower color preferences across the studied range could be merely reflecting the arbitrary livestock presence on different colored populations. Thus, we analyzed the effect of livestock independently for each population. We analyzed the livestock herbivory within populations (419 plants, between 17 and 64 plants per population), using a generalized linear model (GzLM) equivalent to that used in the case of insect herbivores. The explanatory variables were population, flower color, stalk height, leaf length, the flower color * stalk height interaction and the flower color * leaf length interaction (see Appendix S2). Livestock herbivory was fitted to a Binomial distribution with a logit link function.

#### Effect of mutualist and antagonist interactions on seed production

We used a generalized linear model (GzLM) to analyze the effect of the ecological interactions (pollination, seed predation, insect herbivory and cattle herbivory) that may affect total seed number (the response variable), used as a proxy for plant fitness. Cattle herbivory (absence = 0; presence = 1), pollinator visitation rate (No. visits per minute), escape from seed predation (% fruits not affected by seed predators) and insect herbivory (per-plant percentage of herbivory) were the explanatory variables. We also included the population effect into the model (Table 2). Total seed set was fitted to a Poisson distribution with a log link function; and error distribution, link function and model’s structure were chosen by means of the AICc criterion (Burnham & Anderson, 2002). We used data from 93 plants belonging to 8 populations studied in 2010 (Cebreiro, Ancares, Leitariegos, Torrestío, Ventana, San Isidro, Señales and San Glorio) to perform this model.

**Table 2:** GzLM fitted to analyze *G. lutea* reproductive output (total seed number) and its relationship with the livestock herbivory (0/1), the pollinator visitation rate (No. visits/minute), the escape from seed predation (% fruits not affected by seed predators), insect herbivory (percentage of per-plant herbivory) and the population effect. N, 93 individuals. The statistically significant effects are marked in bold (*P* < 0.05).

Dependent variable	Factor	*B*	Wald Chi-Square	d.f.	*P*
Total seed number	**Cattle Herbivory**	−**0.742**	7.293	1	0.007
	**Pollinator visitation rate**	**0.073**	4.569	1	**0.033**
	Escape seed predation	0.005	2.383	1	0.123
	Insect Herbivory	−0.002	0.293	1	0.588
	**Population**		16.484	7	**0.021**

## Results

### Insect and livestock preferences for flower color

**Figure 2:**
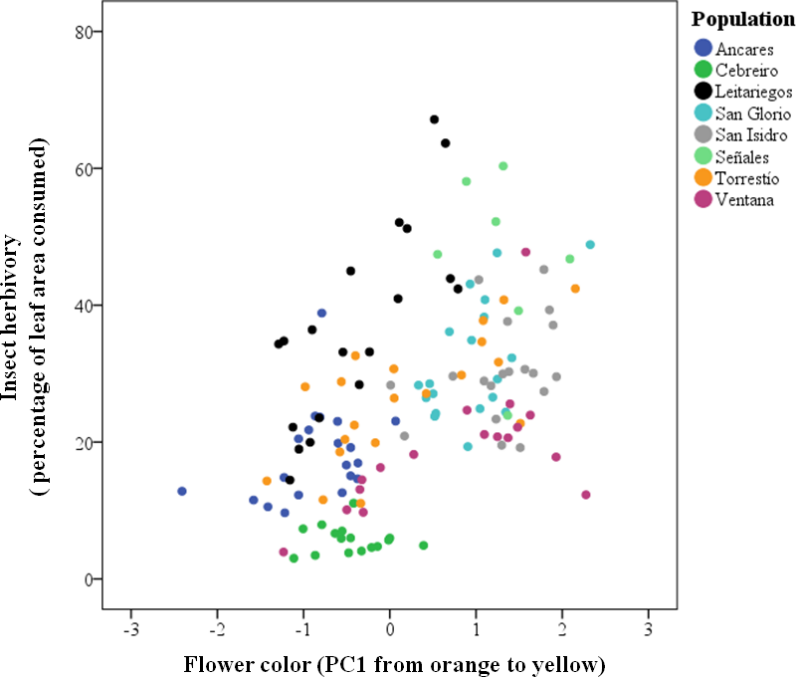
Predicted values of insect herbivory (percentage of eaten leaf area per plant) in relation to *G. lutea* flower color (PC1). N, 104 individuals, from eight populations studied in 2010.

**Figure 3:**
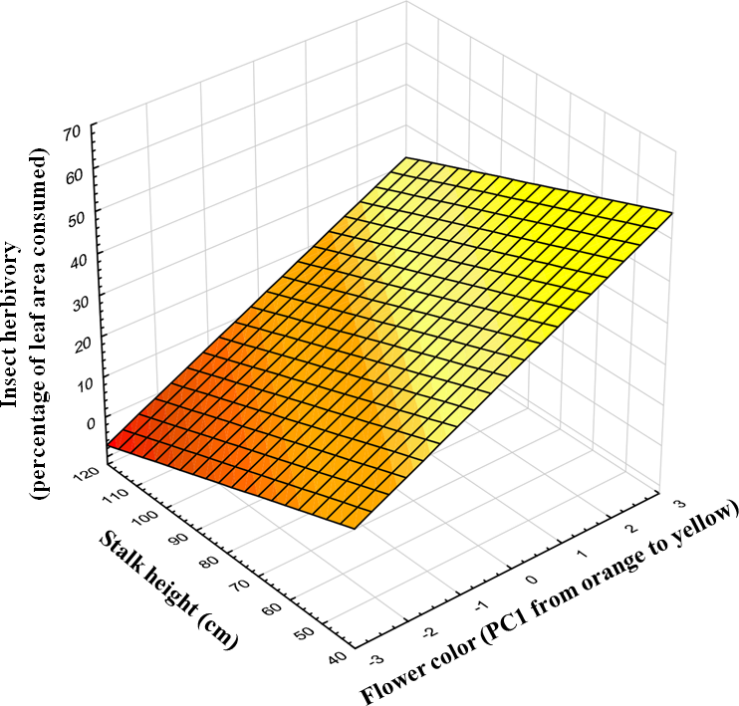
Predicted values of insect herbivory (percentage of eaten leaf area per plant) in relation to flower color (PC1) and correlated stalk height (cm). N, 104 individuals, from 8 populations studied in 2010.

Insect herbivores showed simultaneous preferences for flower color and stalk height when feeding on *Gentiana lutea* (Table 1). Overall, these insects preferred to feed upon yellow-flowering individuals (Fig. 2) and this flower color preference depended on stalk height, as the significant interaction between these two plant traits suggests. Insects prefer yellow-flowering individuals and, among these, shorter individuals were more herbivorized than longer ones (Table 1 and Fig. 3). The relationship between the intensity of herbivory and the interaction between flower color and other plant traits also happens within some of the populations (see Cebreiro, Torrestío and Ventana populations, Appendix S1).

Livestock did not show preferences for flower color within each studied population; although, the probability of livestock herbivory marginally depended on the interaction between flower color and leave length in a population (Cebreiro population; see Appendix S2). Note that significant effects were also not found if the analyses were split by year.

### Effect of mutualists and antagonists on G. lutea reproductive success

After the examination of 63 livestock fecal samples (40 cow samples, 20 horse samples and three sheep samples), we found 13 seeds in six different fecal samples (four cow samples and two horse samples) from four different populations. None of these seeds germinated after the 60 days trial, suggesting that seeds did not survive the digestive track of livestock (a previous study showed 25% germination of *G. lutea* after 45 days, see Losada et al., 2015). Thus, we rule out the seed disperser role of livestock and consider that the interaction with livestock has a negative effect on *G. lutea* reproductive success.

Pollinator visitation rate positively affected plant reproductive output and livestock herbivory decreased seed production. Insect herbivory and seed predation did not affect seed production (Table 2). The most important effect on the *G. lutea* reproductive output turned out to be livestock herbivory, plants which did not suffer livestock herbivory set an average of 5,005 (±416) seeds whereas plants eaten by livestock set an average of 1,269 (±356 seeds). Livestock herbivory affected *G. lutea* reproductive success 10.1 times more than pollinators (see effect sizes, *β* values; Table 2).

## Discussion

Insect herbivores preferred to feed on yellow and short stalk-flowering individuals but livestock did not show flower color preferences along the *Gentiana lutea* range studied (Table 1 and Fig. 2; Appendix S2). In some *G. lutea* populations pollinators and seed predators also prefer the yellow morphs (see Veiga et al., 2015b) but the color preferences vary between populations (see Sobral et al., 2015). Pollinators might visit the more herbivorized individuals in some locations whereas, on other locations, pollinator and herbivores might prefer different corolla colors. This is likely to depend on the pollinator and herbivore community composition as well as on the range of color variation in particular populations.

Floral color in *G. lutea* varies among individuals depending on carotenoids concentration (Zhu et al., 2002). These pigments, which regulate color expression from orange to yellow, are involved in the synthesis of volatile compounds considered to be defenses against insect herbivory (Lakshminarayan, 2013). Alternatively, anthocyanines (which participate in red color expression) may play a dual role, both attracting pollinators and alerting herbivores of a high content of chemical secondary compounds that confer toxicity or, at least make plant tissues difficult to metabolize (Lev-Yadun & Gould, 2009). The fact that insect herbivores preferred to feed upon yellow-flowering individuals might suggest that yellowish corolla color pigments are related to lower amounts of chemical deterrents than orange pigmentation.

Livestock herbivory negatively affected the reproductive success of *G. lutea*, whereas pollination had a positive effect on *G. lutea*’s seed output. We found the effect of livestock herbivory on seed production to be stronger than the effect of pollinators (see *β* values, Table 2). The few studies dealing with the joint effect of herbivores and pollinators on floral traits show that selection by herbivores is often (in 70% of the cases) as strong (or stronger) than selection exerted by pollinators on flowers’ characteristics—herbivores potentially affect floral traits as much as pollinators do (see Johnson, Campbell & Barrett, 2015). Flower color variation in other species is maintained by the balance of selective pressures exerted by mutualisms and antagonisms; for example in *Raphanus sativus* (McCall et al., 2013), *Iris lutescens* (Wang et al., 2013), *Ursinia calenduliflora* (De Jager & Ellis, 2014) and *Geranium thunbergii* (Tsuchimatsu, Yoshitake & Ito, 2014). But, we cannot confirm that either insects or livestock play a role on *G.lutea*’s flower color variation. This is because we found effects of livestock on seed production but not color preferences, and insect herbivores showed color preferences but did not affect seed production. However, insect herbivory also depended on correlations among vegetative traits (such as stalk height or leaf length) and flower color (Table 1; Appendix S1). Thus, if leaf length or stalk height were related to seed production, insects could still be playing an indirect role on the maintenance of flower color variation through pleiotropic effects among correlated plant traits (Fineblum & Rausher, 1997; Herrera et al., 2002; Strauss, Irwin & Lambrix, 2004; Narbona et al., 2014; Johnson, Campbell & Barrett, 2015).

Livestock and insect herbivores do not play a role shaping flower color variation in *G. lutea* (unless through indirect selection). But, pollinators show color preferences and affect *G. lutea*’s fitness. Thus, flower color variation in *G. lutea* might be originated, or at least reinforced, by the selective pressures exerted by pollinators. Flower color variation in polymorphic species may originate from selection by animals which could favor isolation between different color morphs and cause sympatric diversification. However, other reasons could additionally explain flower color variation; for example, the geographic isolation produced by the Quaternary climatic changes has been identified as the main cause of divergence in several mountain plant species (e.g., Martín-Bravo et al., 2010; Alarcón et al., 2012; Blanco-Pastor & Vargas, 2013; Fernández-Mazuecos et al., 2013).

Domesticated animals feeding on *G. lutea* do not show preferences for flower color. Despite it, we argue for the importance of considering the effect of domesticated animals on plant conservation and evolution. Livestock shape plant communities and ecosystems through their interaction with particular species (for example López-Sánchez et al., 2014; López-Sánchez et al., 2015). Human activities are known to have many effects on biodiversity (for example, Dirzo et al., 2014). We might have overlooked a potential indirect effect that human could play on diversity conservation and evolution—through the interactions of domesticated mammals with plant communities. Considering livestock effects on plant communities could better our nature understanding and management.

## Supplemental Information

10.7717/peerj.1685/supp-1 Appendix S1

10.7717/peerj.1685/supp-2 Appendix S2

10.7717/peerj.1685/supp-3 Data S1 Raw Data

## Additional Information and Declarations

### Field Study Permissions

The following information was supplied relating to field study approvals (i.e., approving body and any reference numbers): The Environmental Territorial Service from León, Territorial Delegation of Government of Spain, Regional Government of Castilla and León:12_LE_325_RNA_PuebladeLIllo_INV; Reference: 06.01.013.016/ROT/abp; File number: AEN/LE/103/12.

### Data Availability

The following information was supplied regarding data availability: The raw data is provided as Data S1.
