# Supplementary material for: Flower color preferences of insects and livestock: effects on Gentiana lutea reproductive success
Source: PeerJ. 2016 Mar 15;4:e1685. doi: 10.7717/peerj.1685 (PMC4806593; doi:10.7717/peerj.1685)
Supplement: Appendix S1 [file peerj-04-1685-s001.doc]

**Appendix 1. Insect herbivory (Herbivory Index) and its relationship with flower color and other correlated plant traits, for each of studied populations in 2010.** N = individuals. In bold effects with P < 0.05. Factor codes: LL, leaf length (mm).
