# Supplementary material for: Flower color preferences of insects and livestock: effects on Gentiana lutea reproductive success
Source: PeerJ. 2016 Mar 15;4:e1685. doi: 10.7717/peerj.1685 (PMC4806593; doi:10.7717/peerj.1685)
Supplement: Appendix S2 [file peerj-04-1685-s002.doc]

**Appendix 2. Cattle herbivory results and its relationship with flower color, leaf length, and also the quadratic effect of flower color, for each marked individual from each studied population in 2010 and 2011.**

N = individuals. No effects with P < 0.05. Factor codes: LL, leaf length (mm).
